# Development and validation of algorithms to identify patients with chronic kidney disease and related chronic diseases across the Northern Territory, Australia

**DOI:** 10.1186/s12882-022-02947-9

**Published:** 2022-09-23

**Authors:** Winnie Chen, Asanga Abeyaratne, Gillian Gorham, Pratish George, Vijay Karepalli, Dan Tran, Christopher Brock, Alan Cass

**Affiliations:** 1 Menzies School of Health Research, Charles Darwin University, PO Box 41096, Casuarina, NT 0811 Australia; 2 Royal Darwin Hospital, Darwin, NT Australia; 3 Alice Springs Hospital, Alice Springs, NT Australia

**Keywords:** Chronic kidney disease, Chronic diseases, Diabetes, Diagnostic accuracy, Electronic health records, Electronic phenotype, Hypertension, Validation

## Abstract

**Background:**

Electronic health records can be used for population-wide identification and monitoring of disease. The Territory Kidney Care project developed algorithms to identify individuals with chronic kidney disease (CKD) and several commonly comorbid chronic diseases. This study aims to describe the development and validation of our algorithms for CKD, diabetes, hypertension, and cardiovascular disease. A secondary aim of the study was to describe data completeness of the Territory Kidney Care database.

**Methods:**

The Territory Kidney Care database consolidates electronic health records from multiple health services including public hospitals (*n* = 6) and primary care health services (> 60) across the Northern Territory, Australia. Using the database (*n* = 48,569) we selected a stratified random sample of patients (*n* = 288), which included individuals with mild to end-stage CKD. Diagnostic accuracy of the algorithms was tested against blinded manual chart reviews. Data completeness of the database was also described.

**Results:**

For CKD defined as CKD stage 1 or higher (eGFR of any level with albuminuria or persistent eGFR < 60 ml/min/1.73^2^, including renal replacement therapy) overall algorithm sensitivity was 93% (95%CI 89 to 96%) and specificity was 73% (95%CI 64 to 82%). For CKD defined as CKD stage 3a or higher (eGFR < 60 ml/min/1.73^2^) algorithm sensitivity and specificity were 93% and 97% respectively. Among the CKD 1 to 5 staging algorithms, the CKD stage 5 algorithm was most accurate with > 99% sensitivity and specificity. For related comorbidities – algorithm sensitivity and specificity results were 75% and 97% for diabetes; 85% and 88% for hypertension; and 79% and 96% for cardiovascular disease.

**Conclusions:**

We developed and validated algorithms to identify CKD and related chronic diseases within electronic health records. Validation results showed that CKD algorithms have a high degree of diagnostic accuracy compared to traditional administrative codes. Our highly accurate algorithms present new opportunities in early kidney disease detection, monitoring, and epidemiological research.

**Supplementary Information:**

The online version contains supplementary material available at 10.1186/s12882-022-02947-9.

## Introduction

Globally, the social and economic burden of chronic kidney disease (CKD) is high [1]. The COVID-19 pandemic has brought challenges to the traditional model of episodic, face-to-face care. This has accelerated the adoption of electronic health record (EHR)-based technologies to facilitate virtual models of kidney care [2] – such technologies include clinical decision support tools, and remote disease monitoring platforms for CKD and acute kidney injury. “Electronic phenotype” algorithms are the means through which routinely collected EHR data can be unlocked for secondary use in clinical care [3, 4]. Electronic phenotype algorithms are computerised algorithms that classify patients as disease positive or negative, based on clinical characteristics found within an individual’s existing EHR profile [5, 6]. Typical data elements used in phenotype algorithms include administrative codes, medication classes, and laboratory values [7].

Early EHR research in nephrology relied solely on administrative codes, such as International Classification of Disease (ICD) diagnostic codes; however, administrative codes have limited sensitivity in CKD due to the silent nature of early disease, and clinician under-recognition of the condition [7]. On the other hand, using laboratory cut-off definitions of CKD can be oversensitive compared to manual nephrologist chart reviews [8]. Contemporary CKD phenotype algorithms use a combination of administrative codes and laboratory values to improve diagnostic accuracy [8–13]. Improvements to algorithm accuracy signifies a “critical first step” to advancing kidney care [14] and allows for rapid identification of patients with CKD across health services.

Several CKD phenotype algorithms have been published in recent years – Table 1 provides a summary of key CKD algorithm features and validation results. Published CKD algorithms primarily differ from one another on eGFR cut-offs used to define CKD, proteinuria measures used, and whether their CKD phenotype definition includes or excludes patients on renal replacement therapy (RRT). Algorithm validity refers to the diagnostic sensitivity and specificity of algorithm-classified diagnosis, compared with clinician chart reviews [5, 15]. The plurality of CKD algorithms demonstrate a lack of consensus on a single, “standard” phenotyping approach [16]. There are several reasons for this – firstly, algorithm logic is rarely executed uniformly across healthcare settings due to a lack of standardisation in EHR data structures and coding systems across proprietary vendors [17]; secondly, CKD guidelines and diagnostic criteria differs across countries; thirdly, algorithm requirements differ according to purpose – for example, a CKD phenotype algorithm designed for research recruitment may be unsuitable for use in clinical decision support. Given the context-specific nature of phenotype algorithms, we sought to develop and implement chronic disease algorithms suitable for clinical use within our context in the Northern Territory, Australia.

**Table 1:** Published CKD phenotype algorithms and validation results

**Study**	**CKD phenotype definition**^a^ **(inclusion criteria)**	**Sensitivity for CKD**	**Specificity for CKD**	**Details**
Ernecoff et al., 2019 [12]	CKD stage 4 and 5 including RRT (eGFR < 30 ml/min /1.73m^2^ for duration > 3 months^b^)	100%	0%^c^	Defined as case positive if has a coded diagnosis (ICD-9 or ICD-10) associated with late-stage CKD or RRT, or meets eGFR laboratory criteria
Frigaard et al., 2019 [8]	CKD stages 3a to 4 (eGFR 15-59 ml/min /1.73m^2^ for duration > 3 months)	100%	0%^c^	Defined as case positive on eGFR laboratory criteria only
Nadkarni al, 2014 [10]	CKD stage 3a to 5 including RRT (eGFR < 60 ml/min /1.73m^2^ for duration > 3 months)	93%	96%	Defined as case positive if has a coded diagnosis (ICD-9) associated with CKD and RRT, or meets eGFR laboratory criteria
Norton et al., 2019 [11]	CKD stage 3a to 5 excluding RRT (eGFR < 60 ml/min /1.73m^2^ for duration > 3 months, and/or uACR > 30 mg/g for duration > 3 months) Separate RRT phenotype algorithm described	99%	99%	Defined as case positive for CKD if meets eGFR and/or proteinuria criteria. Other proteinuria measures (urine albumin, urine protein-to-creatinine ratio) used where uACR was unavailable Defined as case positive for RRT if has diagnostic or procedural codes (ICD-9, ICD-10, CPT) associated with RRT Sensitivity and specificity also available for RRT phenotypes
Shang et al., 2021 [13]	CKD stage 1 to 5 excluding RRT (KDIGO G-stage based on eGFR cut-offs for duration > 3 months, A-stage based on uACR or 24-h urine results for duration > 3 months) Separate RRT phenotype algorithm described	87%	97%	Defined as case positive if has a coded diagnosis (ICD-9, ICD-10, CPT, SNOMED) associated with CKD, or meets eGFR and proteinuria laboratory criteria. Other proteinuria measures (urine albumin, urine protein-to-creatinine ratio) used where urine ACR was unavailable Defined as case positive for RRT if has a coded diagnostic or procedural codes associated with RRT Sensitivity and specificity available for pooled CKD phenotype only; no true negatives in validation cohort for individual CKD stages

*Abbreviations*: *eGFR* estimated glomerular filtration rate, *ICD* International Classification of Disease, *CPT* Current Procedural Terminology, *KDIGO* Kidney Disease Improving Global Outcomes, *RRT* Renal replacement therapy, *SNOMED* Systematized Nomenclature of Medicine Clinical Terms, *uACR* urine albumin-to-creatinine ratio

^a^Chronic kidney disease stage as per KDIGO definition

^b^“For duration > 3 months” in Table 1 refers to 2 or more values that meet the eGFR criteria

^c^Calculated from raw data presented in paper, specificity 0% due to no true negatives in validation cohort

The overall objective of the Territory Kidney Care (TKC) project is to improve care for people with CKD in the Northern Territory. Here, we describe the development and validation of chronic disease algorithms to enable region-wide EHR-based initiatives in quality improvement and clinical decision support. Development of our algorithms initially focussed on CKD but subsequently expanded to several commonly co-morbid conditions including type 2 diabetes mellitus (T2DM), hypertension, and cardiovascular disease. Phenotype algorithms rely on secondary use of available EHR data and as such, EHR data quality affects algorithm performance. Previous authors have called for EHR data quality to be reported alongside validation work [5, 16] – for this reason, a secondary aim of the study was to describe data completeness of the TKC database.

## Methods

### Algorithm development

The Territory Kidney Care project began in 2017. The scope of the overall project included 1) linking multiple EHR data sources across the Northern Territory into a consolidated TKC database; 2) developing algorithms to identify patients with CKD and related chronic disease; 3) building a user-interface that utilises algorithm outputs for clinical decision support; and 4) working with health service partners to implement clinical decision support into routine individual-level and service-level care. In this paper, we focus on the algorithm development and validation component of the TKC project. We used an “Agile” approach to algorithm development – undertaking continuous short cycles of guideline consultations, testing, and adaptations to meet user needs [18]. In 2020, the chronic disease algorithms underwent formal face validation as a part of the clinical decision support implementation process. We consulted clinicians within the research team and a panel of local specialists external to the project. The clinicians involved in face validation included 4 nephrologists, 1 endocrinologist, 1 cardiologist, 1 general practitioner, 1 renal nurse, and 1 health informatics nurse working across the Northern Territory. The panel of local clinicians met and reached consensus through discussion on the agreed evidence base, logic of algorithms, and key algorithm assumptions.

Key assumptions for our CKD diagnostic algorithm are outlined in Table 2. The CKD algorithm assigns patients to a CKD stage according to Kidney Disease: Improving Global Outcomes (KDIGO) guidelines [19], according to estimated glomerular filtration rate (eGFR) for G-staging and urine albumin-to-creatinine ratio (uACR) for A-staging of CKD. To fulfill the criteria for a CKD diagnosis based on eGFR, 2 or more readings of persistently reduced eGFR at least 3 months apart was required. The Chronic Kidney Disease Epidemiology Collaboration (CKD-EPI) equation was used for eGFR calculations. Other data elements used included administrative coding from International Classification of Diseases Australian Modified (ICD-10 AM) [20] and primary care International Classification of Primary Care (ICPC-2) codes [21]. Patients were identified as CKD pooled phenotype positive if they had CKD of any stage, or had evidence of renal replacement therapy (RRT) based on administrative codes or ICD procedural codes for RRT.

**Table 2:** Key assumptions for CKD phenotype algorithm

1. Fulfills eGFR^a^ and/or uACR criteria for CKD sub-phenotype stages 1 to 5 according to KDIGO definitions (Supplemental Table 1)
**OR**
2. Has one or more: administrative code or procedural codes criteria for RRT (Supplemental Table 1)
**OR**
3. Has one or more: other administrative codes related to CKD (e.g. chronic glomerulonephritis)

*Abbreviations*: *eGFR* estimated glomerular filtration rate, *ICD-10-AM* International Classification of Disease Australian Modified, *KDIGO* Kidney Disease: Improving Global Outcomes, *RRT* Renal replacement therapy, *uACR* urine albumin-to-creatinine ratio

^a^To fulfill the criteria for CKD based on eGFR, 2 or more readings of persistently reduced eGFR at least 3 months apart was required

**Fig. 1:**
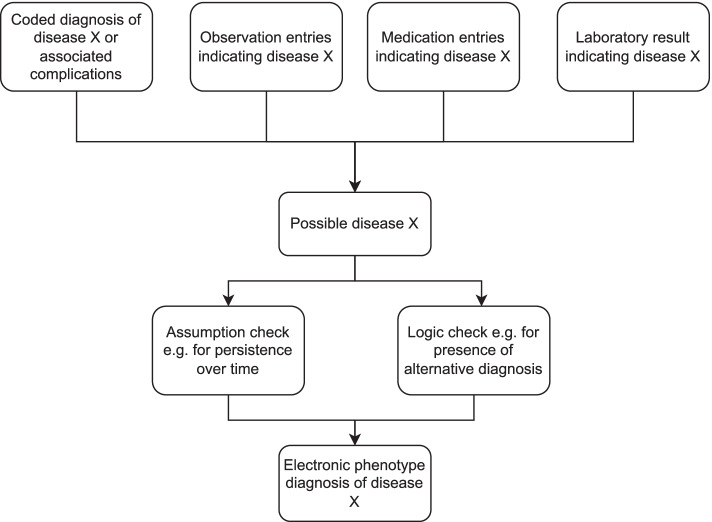
General schema of algorithm logic for chronic disease phenotyping

**Fig. 2:**
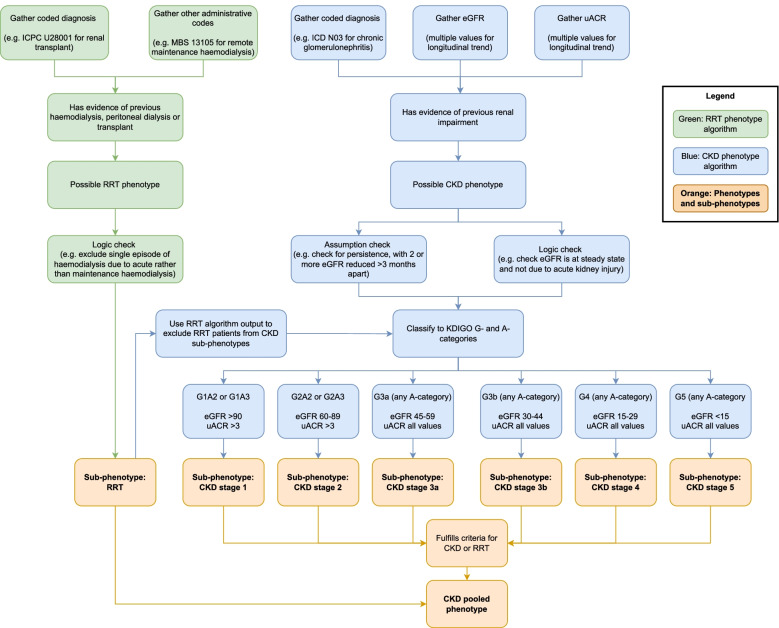
Algorithm logic for chronic kidney disease and renal replacement therapy phenotyping. Abbreviations: eGFR – estimated glomerular filtration rate; ICD – International Classification of Disease; ICPC – International Classification of Primary Care; KDIGO – Kidney Disease: Improving Global Outcomes; MBS – Medicare Benefits Scheme; RRT – Renal replacement therapy; uACR – urine albumin-to-creatinine ratio

A similar algorithm logic approach was used for related chronic diseases including T2DM, hypertension, and cardiovascular disease. Figure 1 shows a simplified general schema of our chronic disease algorithm logic and Fig. 2 demonstrates how the algorithm logic was applied specifically to CKD and RRT phenotype algorithms. Full details and executable code of our chronic disease algorithms are publicly available online [22].

### Setting and study population

We applied the chronic disease algorithms to the TKC database. The TKC database is conceptually similar to a EHR-based CKD registry. The development of this region-wide database was a substantial undertaking – geographically, the Northern Territory covers an area of approximately 1.4 million km^2^; from an EHR point of view, the database consolidates siloed EHR systems across all public hospitals (*n* = 6), all publicly-funded remote primary health care clinics (*n* = 56), and participating non-government primary health care services (*n* = 12) in the Northern Territory. Individual records from each health service are mapped and linked prior to phenotype algorithm execution. The consolidated database includes adults with CKD or a risk factor for CKD and has up to 24 years span of longitudinal data (1998 to 2022). Adults at risk of CKD included patients with pre-existing diabetes and hypertension, a history of renal disease or acute kidney injury, and patients with a high cardiovascular risk score (Framingham five-year cardiovascular risk > 15%).

As of 07 February 2021, there were *n* = 48,569 patients within the TKC database who were active – active is defined as patients with a TKC database entry within the past 2 years. A stratified random sample of active patients with various chronic diseases, including mild to end-stage CKD, was selected for validation (total *n* = 360). All patients had to have 3 or more laboratory and observation entries to be considered for inclusion. Six subgroups of patients were selected to ensure that the validation cohort included both algorithm positive cases, and algorithm negative controls for each of the chronic diseases of interest (CKD, diabetes, hypertension, cardiovascular disease). Subgroup criteria are described in Table 3. Briefly, subgroup 1 were patients at risk of CKD with no known disease; subgroups 2, 3 and 4 were patients in mild, moderate and severe CKD stages; and subgroups 5 and 6 were patients with comorbidities (e.g. diabetes) with or without CKD. Subgroup selection was based on CKD stages or co-morbidities, as defined by algorithm outputs. A random number generator selected *n* = 60 patients within each of the 6 subgroups.

**Table 3:** Subgroup criteria for validation cohort

**Subgroup**	**Inclusion criteria**^a^	**Number in final validation cohort (total *n* = 288)**
Subgroup 1	Patients at risk of CKD, with no known diagnosis of CKD	*n* = 50
Subgroup 2	Patients with CKD stages 1 to 3a	*n* = 49
Subgroup 3	Patients with CKD stages 3b to 4	*n* = 51
Subgroup 4	Patients with CKD stage 5 or on renal replacement therapy	*n* = 50
Subgroup 5	Patients with 2 or more coded ICD/ICPC co-morbidities (diabetes, hypertension, cardiovascular disease)	*n* = 45
Subgroup 6	Patients with 3 or more medications for chronic disease (diabetes, hypertension, cardiovascular disease medications), with or without CKD	*n* = 43

*Abbreviations*: *ICD* International Classification of Disease, *ICPC* International Classification of Primary Care

^a^CKD stages and the presence of comorbidities was based on algorithm output

### Chart reviews

Algorithm generated diagnoses were compared against blinded clinician reviews of de-identified patient charts. Pilot testing of a smaller sample of patients (*n* = 120) was conducted. Five physicians across nephrology, internal medicine, and general practice participated in the study (WC, PG, JK, DT, CB). Inform consent was obtained from clinician participants. Each reviewer was assigned a random subset of the validation cohort, which contained a mix of patients from each of the subgroups. Two independent clinicians reviewed all administrative codes, medications, observations, laboratory results and other structured data available in the TKC database, via a front-end user interface. Clinicians had access to text search and result visualisation functions within the front-end interface. Identifiable patient demographic information (name, date of birth, health record number) was masked, and participants were blinded to algorithm generated diagnoses. Clinicians recorded their diagnoses for CKD staging according to KDIGO definitions, and presence or absence of diabetes, hypertension, and cardiovascular disease using a structured tool. Discordant diagnoses between the two clinicians were resolved by consensus with reference to the agreed evidence base and by a third clinician where consensus could not be reached. For example, the agreed evidence base at the time of clinician manual review included the 2012 KDIGO guidelines for diagnosing CKD [19] and the 2016 Australian Heart Foundation guidelines for hypertension [23]. The study was completed within a four-week timeframe in February 2021.

### Sample size

Using the Buderer formula for calculating sample size for diagnostic accuracy testing [24], a sample of *n* = 277 patient records was required to obtain a margin of error of ± 5% for sensitivity and specificity. This is based on an expected sensitivity of 95%, specificity of 90%, prevalence of disease set at 50%, and an alpha of 0.05. Sensitivity and specificity estimates were based on pilot testing results.

### Analysis

Algorithm diagnoses were compared against chart reviews as the reference gold standard. Sensitivity and specificity of each chronic disease and 95% confidence intervals (asymptotic method) were reported. Overall accuracy for the overall CKD algorithm (CKD of any stage) and accuracy of CKD staging algorithms (CKD sub-phenotypes for CKD stages 1 to 5, and RRT) were reported. For validation, RRT sub-phenotype was considered mutually exclusive to all other CKD stages. We conducted a sensitivity analysis with 1) CKD phenotype defined as KDIGO stage 3a and above, which is the main definition of CKD used in previous studies (Tables 1 and 3) CKD phenotype algorithm using a more stringent uACR criteria of two or more elevated readings over > 3 months. Accuracy of administrative codes (ICD/ICPC) was also compared to that of clinician chart reviews (gold standard). For EHR data quality, we used the domains of assessing data completeness proposed by Wieskopf et al. [25] – descriptive statistics were reported for documentation, breadth, and density of the data within the TKC database. The proportion of patients within the database meeting several data completeness metrics were reported. Analysis was conducted using Stata version 15.1 (StataCorp, 2017) [26] and R (R Core Team 2021) [27].

### Ethics approval

The Human Research Ethics Committee of the Northern Territory Department of Health and Menzies School of Health Research (HREC-2020–3903) and the Central Australian Human Research Ethics Committee (CA-20–3919) approved the study protocol.

## Results

### Overview

**Fig. 3:**
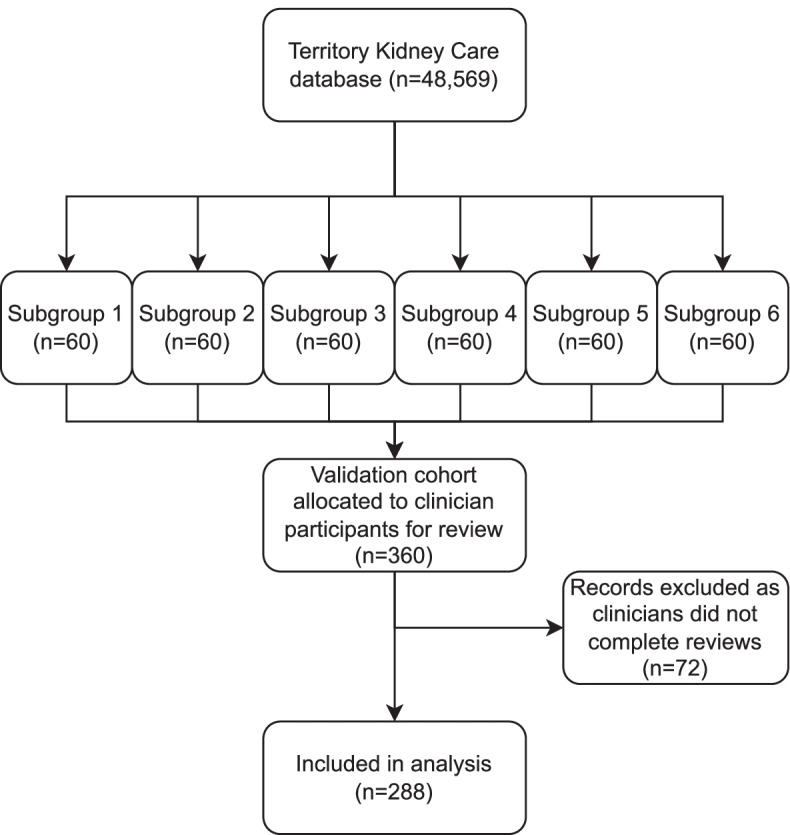
Flowchart of the validation cohort

A total of *n* = 360 patients were selected for the validation cohort and assigned to 7 clinician participants. Due to 2 clinician participants not completing their assigned records for review within the study timeframe, *n* = 72 patients were excluded from analysis. Five clinician participants conducted two independent chart reviews for *n* = 288 patient files (Fig. 3). Table 3 shows the number of records reviewed in each subgroup.

For the chart reviewed patients, median age was 46 (IQR 33 to 57) and 44% were male. Other demographic information is included in Table 4. The average time taken for clinicians to complete a structured chart review within the TKC database was 2.24 min and total time taken for all chart reviews in the validation cohort was approximately 23 h. Inter-rater reliability was high – raw percentage agreement values were between 83 and 94%; and Cohen’s Kappa between 0.66 to 0.86 for each chronic disease (see Supplemental Table 2).

**Table 4:** Basic demographics of included chart review patients

**Demographic**^a^	**Chart reviewed patients** **Total *n* = 288**
	**Median (IQR) or N (%)**
Age	46 (33 to 57)
Sex – male	127 (44%)
Sex – female	161 (56%)
CKD mild to moderate (1 to 3a)	180 (63%)
CKD moderate to severe (3b to 5)	68 (24%)
RRT	40 (14%)
T2DM	80 (28%)
Hypertension	143 (50%)
Cardiovascular disease	77 (27%)

*Abbreviations*: *RRT* Renal replacement therapy, *T2DM* Type 2 diabetes mellitus

^a^Chronic disease prevalence as per clinician chart review diagnoses

### Accuracy of CKD phenotypes

Algorithm validation results are presented in Table 5. Overall algorithm sensitivity for CKD pooled phenotype defined as CKD stage 1 or higher (including RRT) was 93% (95%CI 89 to 96%), with a specificity of 73% (95%CI 64 to 82%)). In the sensitivity analysis (Table 6), algorithm sensitivity remained the same (93%), but specificity improved markedly (97%) when CKD phenotype was defined as CKD stage 3a or higher. When albuminuria was defined using the more stringent criteria of 2 or more elevated uACR readings at least 3 months apart, CKD algorithm specificity increased to 94% but sensitivity dropped to 88%.

**Table 5:** Accuracy of algorithm diagnosis and administrative code diagnosis, versus clinician diagnosis (gold standard)

**Phenotype or sub-phenotype**	**TKC algorithm**		**Coded diagnosis (ICD/ ICPC)**^a^	
	**Sensitivity (%, 95%CI)**	**Specificity (%, 95%CI)**	**Sensitivity (%, 95%CI)**	**Specificity (%, 95%CI)**
CKD any stage (CKD 1 or higher)	93% (89 to 96%)	73% (64 to 82%)	72% (66 to 78%)	97% (93 to 100%)
CKD stage 1	87% (76 to 98%)	90% (87 to 94%)	29% (15 to 43%)	96% (94 to 98%)
CKD stage 2	70% (56 to 84%)	98% (96 to 99%)	30% (16 to 44%)	91% (87 to 94%)
CKD stage 3a	70% (42 to 98%)	100% (99 to 100%)	70% (42 to 98%)	95% (93 to 98%)
CKD stage 3b	82% (70 to 95%)	99% (98 to 100%)	15% (3 to 27%)	98% (97 to 100%)
CKD stage 4	70% (50 to 90%)	99% (98 to 100%)	30% (10 to 50%)	98% (97 to 100%)
CKD stage 5	100% (100 to 100%)	100% (99 to 100%)	21% (0 to 43%)	100% (100 to 100%)
RRT	100% (100 to 100%)	98% (96 to 100%)	100% (100 to 100%)	98% (96 to 100%)
T2DM	75% (66 to 85%)	97% (94 to 99%)	95% (90 to 100%)	91% (87 to 95%)
Hypertension	85% (80 to 91%)	88% (83 to 94%)	76% (68 to 83%)	90% (86 to 95%)
Cardiovascular disease	79% (70 to 88%)	96% (94% to 99%)	N/A	N/A

Abbreviations: *CI* Confidence interval, *ICD* International Classification of Disease, *ICPC* International Classification of Primary Care, *RRT* Renal replacement therapy, *T2DM* Type 2 diabetes mellitus, *TKC* Territory Kidney Care

^a^For CKD staging, where ICD/ICPC differed, the average CKD stage of the two were taken, rounded up to the nearest integer. For cardiovascular disease TKC algorithms used ICD/ICPC codes only

**Table 6:** CKD algorithm sensitivity analysis

CKD phenotype definition	Sensitivity (%, 95%CI)	Specificity (%, 95%CI)
CKD defined as stage 1 or higher (eGFR < 60 ml/min/1.73^2^ and/or persistent urine albuminuria, including RRT)	93% (89 to 96%)	73% (64 to 82%)
CKD defined as stage 3a or higher (eGFR < 60 ml/min/1.73^2^, including RRT)	93% (89 to 98%)	97% (94 to 99%)
CKD defined as stage 1 or higher, requiring 2 or more elevated uACR > 3 months apart^a^	88% (83 to 92%)	94% (88 to 99%)

*Abbreviations*: *CI* Confidence interval, *eGFR* Estimated glomerular filtration rate, *RRT* Renal replacement therapy

^a^2 or more elevated uACR required for diagnosis of CKD stage 1 and CKD stage 2 only

**Fig. 4:**
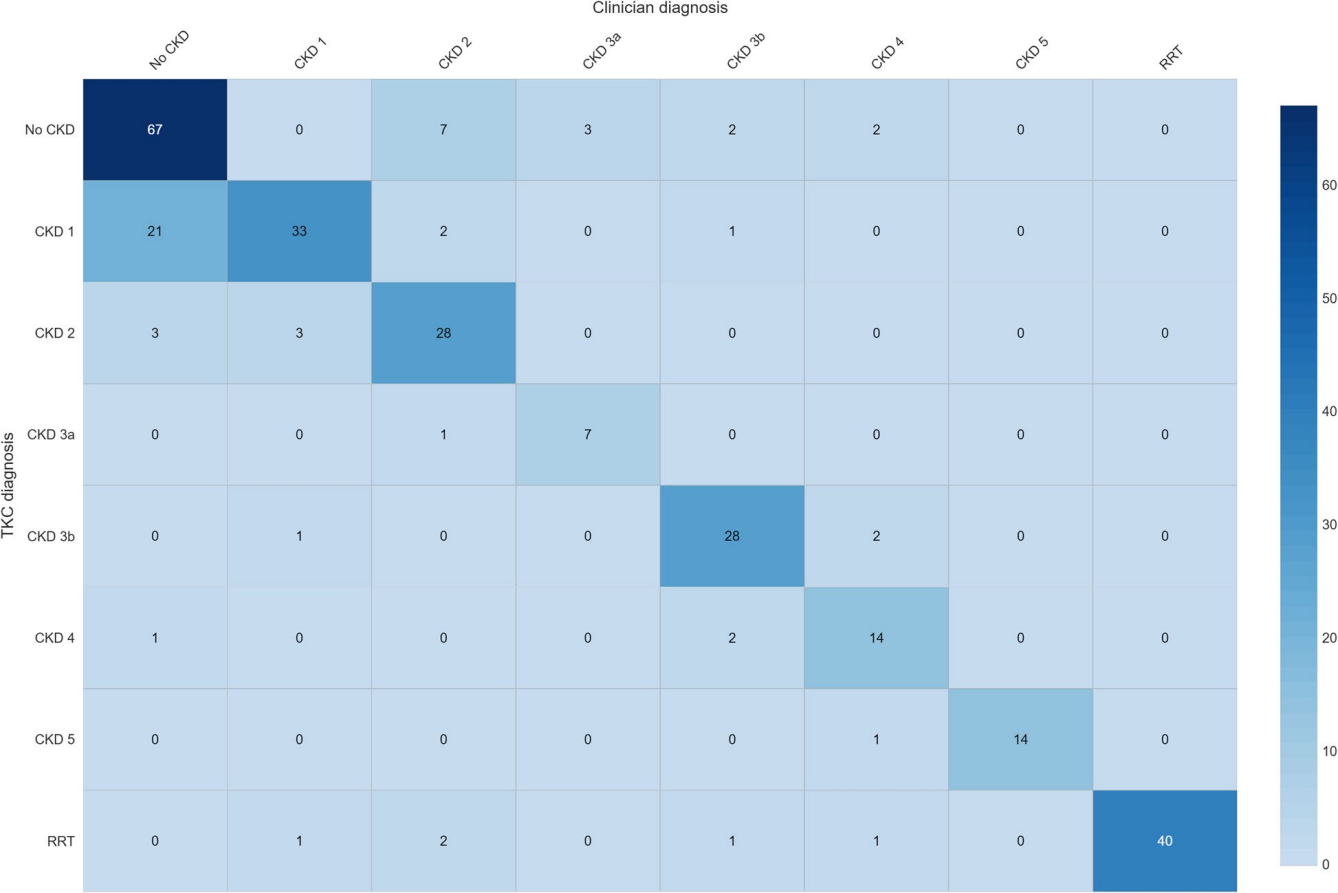
Confusion matrix for algorithm versus clinician diagnosis*. Abbreviations: RRT – Renal replacement therapy; TKC – Territory Kidney Care (algorithm). *Cells indicate total number of patients (n) in each category, clinician diagnosis (gold standard) versus TKC algorithm diagnosis

A confusion matrix for CKD staging is seen in Fig. 4 – reasons why TKC algorithms differed from clinician diagnoses included the presence of acute kidney injury and episodic haemodialysis (e.g. patients previously on maintenance haemodialysis but with no recent episodes), wide fluctuations in eGFR readings, and limited availability of laboratory data. Algorithms applied strict laboratory diagnostic definitions for CKD staging whereas clinicians had variable guideline interpretation where objective data was insufficient to reach a clear diagnostic conclusion. For example, TKC algorithms would classify a patient with a single elevated uACR and one eGFR between 60–89 as “no CKD” (G2A0 as no disease, according to KDIGO guidelines), whereas clinicians may classify the same patient as having CKD stage 2 despite not strictly meeting the KDIGO persistence criteria for a diagnosis of CKD [19]. Administrative codes (ICD/ICPC) were less sensitive than TKC algorithms at diagnosing CKD (72 vs 93%) but had higher specificity (97 vs 73%). For CKD sub-phenotypes, the algorithms consistently outperformed administrative codes – algorithm sensitivity for individual CKD stages (70.00 to 100%) was substantially higher than that of ICD/ICPC coded diagnoses (15 to 100%). Specificity of algorithms and ICD/ICPC codes was similarly high, at 90% or above for all CKD sub-phenotypes. Notably, ICD/ICPC coded diagnoses of CKD stage 5 without RRT had very low sensitivity compared to algorithm sensitivity (21 vs 100%). Examples where ICD/ICPC coded diagnoses missed CKD stage 5 cases included patient records where eGFR drop was recent, or in cases where patients had recently started RRT.

### Accuracy of other chronic disease phenotypes

For related chronic diseases, the T2DM algorithm had a sensitivity of 75% (95%CI 66 to 85%) and specificity of 97% (95%CI 94 to 99%); hypertension algorithm had a sensitivity of 85% (95%CI 80 to 91%) and specificity of 88% (95%CI 83 to 94%); and cardiovascular disease had a sensitivity of 79% (95%CI 70 to 88%) and specificity of 96% (95%CI 94 to 99%). Differences between TKC algorithm and clinician diagnoses occurred where diagnostic codes and objective measures were not concordant. As with CKD, TKC algorithms generally applied a stricter definition of disease than clinicians. For example, the algorithm required 2 or more elevated HbA1c for a diagnosis of diabetes – hence patients with a single historic elevated HbA1c reading and several normal range HbA1c readings, with no other evidence of diabetes (e.g. no glucose-lowering medications) is algorithm coded as “no diabetes”. For full sensitivity, specificity, positive predictive value (PPV), negative predictive value (NPV), and area under ROC curve results see Supplemental materials.

### Data completeness metrics

As of 07 February 2021, there were *n* = 48,569 patients in the TKC database who had an active entry within the last 2 years. Median timespan between first and last data entry for a single patient was 11 years (IQR 2–18). Data metrics of all active patients are displayed in Supplemental Tables 3 and 4. The highest number of patients had a medication entry compared to other data types (94%). Approximately two-thirds of patients had a recorded ICPC code, ICD code, observation entry or laboratory result. Out of the five data types, laboratory results had the highest median number of results per patient (*n* = 116, IQR 40 to 261) and greatest median density of results per patient (*n* = 9.0, IQR 4.0 to 18.6). Four metrics were used to report data completeness. Metric 4 had the most stringent criteria for data completeness (3 laboratory results, 3 observation entries, 1 coded diagnosis, and 1 medication entry) and this minimum requirement for data completeness was met in 61% of individual patient files.

## Discussion

Algorithm-assisted disease identification is gaining momentum in nephrology [2, 3]. Accurate, validated algorithms are fundamental to EHR-based innovations in early CKD detection, intervention, and monitoring [14, 28]. To our knowledge, this is the first published study describing diagnostic sensitivity and specificity for all CKD sub-phenotypes from stages 1 to 5, through to RRT. Despite a growing volume of EHR-based research and EHR-based clinical decision support tools, validation can at times be seen as “a mere poor relative of the real original research” [29]. Few rigorous validation studies have been conducted outside of large established phenotype collaborations such as the eMERGE Network [30].

Our results showed that CKD algorithms consistently outperformed administrative codes (ICD/ICPC) in correctly classifying patients into individual CKD stages. The poor sensitivity of administrative codes was particularly striking for CKD stage 5 – the implication of this is that ICD/ICPC codes alone are unreliable for EHR-based detection of late-stage CKD. Our highly accurate CKD staging algorithms unlocks new opportunities for personalised care. For example, the algorithm outputs have been used in the TKC project to drive clinical decision support alerts that identify and target interventions for patients with rapidly progressing CKD across our region. These validated algorithms are also useful for population-level disease progression monitoring and EHR-based epidemiological research.

CKD validation studies to date have primarily considered CKD as a single pooled disease phenotype (Table 1). Only one study in 2021 considered CKD sub-phenotypes in their validation – however, a limitation of this study by Shang et al. was that sensitivity and specificity was reported for the pooled CKD phenotype but not for CKD sub-phenotypes (CKD stages 1 to 5) [13]. We used a similar pooled definition of CKD to Shang et al., defining CKD as KDIGO stage 1 or higher. In contrast, most other CKD validation studies defined CKD as KDIGO stage 3a or higher (eGFR < 60 ml/min/1.73m2) – using this common definition of CKD, our algorithm had a sensitivity of 93% and specificity of 97%, and was comparable to existing studies with sensitivities ranging from 93 to 100% and specificities ranging from 0 to 99% [8, 10, 11]. Our algorithm sensitivity and specificity for diabetes [31–33], hypertension [34, 35], and cardiovascular disease [36, 37] also have comparable accuracy to that of previously published studies.

Evident in several CKD algorithm validation studies is the problem of “0%” specificity [8, 12]. To reduce time burden on clinicians, chart reviews may be limited to individuals who are algorithm positive for CKD. However, where there are no true negatives in the validation cohort, 0 is the numerator for the specificity equation, resulting in a specificity of 0% (specificity = true negative / true negative and false negatives). We encountered this problem during our pilot study, but overcame the issue through selection of an appropriate true negative population in our validation cohort – appropriate true negatives being patients with risk factors for CKD but no known kidney disease.

### Strengths and limitations

A strength of this study was the number of CKD and related chronic disease algorithms validated for clinical use. Only key algorithms were selected for the purpose of validation, but we developed a large number of algorithms to classify patients into additional nuanced CKD sub-phenotypes according to operational requirements – for example, CKD sub-phenotypes based on mode of RRT (e.g. haemodialysis or transplant sub-phenotype), and sub-phenotypes based on KDIGO G and A-staging (e.g. CKD G2A2 and G2A3). We recognised a need to move beyond the quest for an ideal CKD algorithm – therefore, we tested several adaptations of our CKD algorithm and conducted a sensitivity analysis to quantify sensitivity and specificity trade-offs of minor adjustments to the CKD phenotype definition. Our validation study was adequately powered to ensure precision of accuracy results. The TKC algorithm utilised EHR from diverse health services to improve data element availability [17]. For example, where previous CKD phenotypes used proxy measures for albuminuria (e.g. urinalysis results) due to low uACR availability [11, 13] our broad coverage of EHR sources across the Northern Territory, including laboratory results from primary care, allowed us to achieve CKD A-staging directly from uACR values; in our study, at least 1 urine ACR was available in 40% of active patients in the TKC database, compared to 7% urine ACR availability in a previous CKD algorithm validation study [11].

Nevertheless, there is room to expand what and how EHR data is used in our chronic disease algorithms. Several Australian studies described high algorithm accuracy through incorporating keyword searches for chronic diseases within “reason for encounter” fields [32, 33]. These primary care EHR fields are not currently available within the TKC database but a next step of the TKC project is to expand the database to incorporate additional EHR systems used in private general practices and private specialist outpatients across our region. Natural language processing (NLP) for unstructured data extraction from free text and machine learning algorithms have also been incorporated into CKD algorithms [12, 13]. For CKD algorithms, a possible application of NLP would be to extract free-text data within imaging reports to identify structural kidney abnormalities. Despite the increasing popularity of NLP and machine learning [38], using more EHR data elements in algorithms does not guarantee improvements in diagnostic accuracy [12, 39] – we are still investigating how to leverage these techniques to optimise our algorithms. Another limitation of our algorithms and phenotype algorithms more broadly is limited universal portability. Given the heterogeneous nature of vendor-specific EHR data structures and semantic standards, algorithms cannot be directly executed across EHR types without resource-intensive customisation [40, 41].

There are several limitations to our validation methodology. Firstly, we used a stratified random sample to ensure capture of positive and negative cases – thus, our validation cohort is not reflective of the entire TKC database. Algorithm studies like ours typically select a limited sample of the entire database for validation, as manual chart reviews are labour and resource-intensive to conduct. Secondly, algorithm validation studies frequently use clinician chart reviews but lack an objective gold standard “source of truth” [5, 42, 43]. To minimise bias we used two independent, blinded reviewers and achieved a high level of inter-reviewer agreement. Thirdly, our validation period was extended from a planned two-week period to a four-week period due to lack of clinician availability to complete the chart reviews within a shorter timeframe. This introduced a small possibility of discrepancies in the “live” TKC database (e.g. new eGFR results entering the system) between time of clinician manual chart review and time of extraction for TKC algorithm-coded diagnoses. Finally, we reported data completeness metrics but other EHR data quality issues could have affected our validation results.

## Conclusions

As EHR data is increasingly used for secondary purposes, there remains a need for algorithm development and validation. Our study describes the development and validation of algorithms to identify individuals with CKD and related chronic diseases. Validation results demonstrated that CKD staging algorithms have superior sensitivity and specificity compared to administrative codes alone. Our highly accurate CKD staging algorithms facilitates innovations in early kidney disease detection and monitoring, personalised clinical care, and EHR-based epidemiological research.

## Supplementary Information

**Additional file 1.**

## Data Availability

The datasets generated during the study are included in this published article and its supplementary files.

## Abbreviations

CKD: Chronic kidney disease
CKD-EPI: Chronic Kidney Disease Epidemiology Collaboration
CPT: Current Procedural Terminology
eGFR: Estimated glomerular filtration rate
EHR: Electronic health record
ICD: International Classification of Disease
ICPC: International Classification of Primary Care
KDIGO: Kidney Disease Improving Global Outcomes
NPV: Negative predictive value
PPV: Positive predictive value
RRT: Renal replacement therapy
SNOMED: Systematized Nomenclature of Medicine Clinical Terms.
T2DM: Type 2 diabetes mellitus
TKC: Territory Kidney Care
uACR: Urine albumin-to-creatinine ratio

## References

[1] Bikbov B, Purcell CA, Levey AS, Smith M, Abdoli A, Abebe M (2020). Global, regional, and national burden of chronic kidney disease, 1990–2017: a systematic analysis for the Global Burden of Disease Study 2017. Lancet.

[2] Wang C-S, Ku E (2020). eHealth in kidney care. Nat Rev Nephrol.

[3] Glenn D, Gibson KL (2019). Finding that needle in the haystack: computable phenotypes. J Am Soc Nephrol.

[4] Shah SM, Khan RA (2020). Secondary use of electronic health record: opportunities and challenges. IEEE Access.

[5] Richesson R, Wiley L, Gold S, Rasmussen L. Rethinking Clinical Trials: Electronic Health Records-Based Phenotyping USA: NIH Collaboratory; 2021 [cited 2021 November]. Available from: https://rethinkingclinicaltrials.org/chapters/conduct/electronic-health-records-based-phenotyping/.

[6] Richesson RL, Smerek MM, Blake Cameron C (2016). A framework to support the sharing and reuse of computable phenotype definitions across health care delivery and clinical research applications. EGEMS (Wash DC).

[7] Grams ME, Plantinga LC, Hedgeman E, Saran R, Myers GL, Williams DE (2011). Validation of CKD and related conditions in existing data sets: a systematic review. Am J Kidney Dis.

[8] Frigaard M, Rubinsky A, Lowell L, Malkina A, Karliner L, Kohn M (2019). Validating laboratory defined chronic kidney disease in the electronic health record for patients in primary care. BMC Nephrol.

[9] Ostropolets A, Reich C, Ryan P, Shang N, Hripcsak G, Weng C (2020). Adapting electronic health records-derived phenotypes to claims data: Lessons learned in using limited clinical data for phenotyping. J Biomed Inform.

[10] Nadkarni GN, Gottesman O, Linneman JG, Chase H, Berg RL, Farouk S (2014). Development and validation of an electronic phenotyping algorithm for chronic kidney disease. AMIA Annu Symp Proc.

[11] Norton JM, Ali K, Jurkovitz CT, Kiryluk K, Park M, Kawamoto K (2019). Development and validation of a pragmatic electronic phenotype for CKD. Clin J Am Soc Nephrol.

[12] Ernecoff NC, Wessell KL, Hanson LC, Lee AM, Shea CM, Dusetzina SB (2019). Electronic health record phenotypes for identifying patients with late-stage disease: a method for research and clinical application. J Gen Intern Med.

[13] Shang N, Khan A, Polubriaginof F, Zanoni F, Mehl K, Fasel D (2021). Medical records-based chronic kidney disease phenotype for clinical care and “big data” observational and genetic studies. NPJ Digit Med.

[14] Tummalapalli SL, Peralta CA (2019). An electronic CKD phenotype: a step forward in improving kidney care. Clin J Am Soc Nephrol.

[15] Denaxas S, Gonzalez-Izquierdo A, Direk K, Fitzpatrick NK, Fatemifar G, Banerjee A (2019). UK phenomics platform for developing and validating electronic health record phenotypes: CALIBER. J Am Med Inform Assoc.

[16] Chapman M, Mumtaz S, Rasmussen LV, Karwath A, Gkoutos GV, Gao C (2021). Desiderata for the development of next-generation electronic health record phenotype libraries. GigaScience.

[17] Rasmussen LV, Brandt PS, Jiang G, Kiefer RC, Pacheco JA, Adekkanattu P (2020). Considerations for improving the portability of electronic health record-based phenotype algorithms. AMIA Annu Symp Proc.

[18] Holden RJ, Boustani MA, Azar J (2021). Agile Innovation to transform healthcare: innovating in complex adaptive systems is an everyday process, not a light bulb event. BMJ Innovations.

[19] Kidney Disease Improvement Global Outcomes (KDIGO). KDIGO 2012 Clinical Practice Guideline for the Evaluation and Management of Chronic Kidney Disease. Kidney Int Suppl. 2013;3(1):1–150. 10.1038/ki.2013.243 23989362

[20] Independent Hospital Pricing Authority (IHPA). ICD-10-AM/ACHI/ACS current edition 2019 [cited 2021 January]. Available from: https://www.ihpa.gov.au/what-we-do/icd-10-am-achi-acs-current-edition.

[21] World Health Organization. International Classification of Primary Care, Second edition (ICPC-2): WHO; 2003 [cited 2020 July]. Available from: https://www.who.int/classifications/icd/adaptations/icpc2/en/.

[22] Abeyaratne A. Github - TKC Picorules Rules 2020 [cited 2020 December]. Available from: https://github.com/asaabey/tkc-picorules-rules.

[23] National Heart Foundation of Australia. Guideline for the diagnosis and management of hypertension in adults - 2016 Melbourne: National Heart Foundation of Australia; 2016 [cited 2022 January]. Available from: https://www.heartfoundation.org.au/getmedia/c83511ab-835a-4fcf-96f5-88d770582ddc/PRO-167_Hypertension-guideline-2016_WEB.pdf.

[24] Buderer NM (1996). Statistical methodology: I. Incorporating the prevalence of disease into the sample size calculation for sensitivity and specificity. Acad Emerg Med.

[25] Weiskopf NG, Hripcsak G, Swaminathan S, Weng C (2013). Defining and measuring completeness of electronic health records for secondary use. J Biomed Inform.

[26] StataCorp (2017). Stata Statistical Software: Release 15.

[27] R Core Team. R: A Language and Environment for Statistical Computing Vienna, Austria: R Foundation for Statistical Computing; 2021 [cited 2021 November]. Available from: https://www.R-project.org/.

[28] Cameron B, Douthit B, Richesson R (2018). Data and knowledge standards for learning health: A population management example using chronic kidney disease. Learning Health Systems.

[29] Ehrenstein V, Petersen I, Smeeth L, Jick SS, Benchimol EI, Ludvigsson JF (2016). Helping everyone do better: a call for validation studies of routinely recorded health data. Clin Epidemiol.

[30] Gottesman O, Kuivaniemi H, Tromp G, Faucett WA, Li R, Manolio TA (2013). The Electronic Medical Records and Genomics (eMERGE) Network: past, present, and future. Genet Med.

[31] Spratt SE, Pereira K, Granger BB, Batch BC, Phelan M, Pencina M (2017). Assessing electronic health record phenotypes against gold-standard diagnostic criteria for diabetes mellitus. J Am Med Inform Assoc.

[32] Rahimi A, Liaw ST, Taggart J, Ray P, Yu H (2014). Validating an ontology-based algorithm to identify patients with type 2 diabetes mellitus in electronic health records. Int J Med Inform.

[33] Havard A, Manski-Nankervis J-A, Thistlethwaite J, Daniels B, Myton R, Tu K (2021). Validity of algorithms for identifying five chronic conditions in MedicineInsight, an Australian national general practice database. BMC Health Serv Res.

[34] McDonough CW, Babcock K, Chucri K, Crawford DC, Bian J, Modave F (2020). Optimizing identification of resistant hypertension: Computable phenotype development and validation. Pharmacoepidemiol Drug Saf.

[35] Teixeira PL, Wei W-Q, Cronin RM, Mo H, VanHouten JP, Carroll RJ (2017). Evaluating electronic health record data sources and algorithmic approaches to identify hypertensive individuals. J Am Med Inform Assoc.

[36] Liao KP, Ananthakrishnan AN, Kumar V, Xia Z, Cagan A, Gainer VS (2015). Methods to develop an electronic medical record phenotype algorithm to compare the risk of coronary artery disease across 3 chronic disease cohorts. PLoS ONE.

[37] Rubbo B, Fitzpatrick NK, Denaxas S, Daskalopoulou M, Yu N, Patel RS (2015). Use of electronic health records to ascertain, validate and phenotype acute myocardial infarction: a systematic review and recommendations. Int J Cardiol.

[38] Pendergrass SA, Crawford DC (2019). Using electronic health records to generate phenotypes for research. Curr Protoc Hum Genet.

[39] Shivade C, Raghavan P, Fosler-Lussier E, Embi PJ, Elhadad N, Johnson SB (2013). A review of approaches to identifying patient phenotype cohorts using electronic health records. J Am Med Inform Assoc.

[40] Samwald M, Fehre K, de Bruin J, Adlassnig K-P (2012). The Arden Syntax standard for clinical decision support: Experiences and directions. J Biomed Inform.

[41] Loya SR, Kawamoto K, Chatwin C, Huser V (2014). Service oriented architecture for clinical decision support: a systematic review and future directions. J Med Syst.

[42] Berner ES (2003). Diagnostic decision support systems: how to determine the gold standard?. J Am Med Inform Assoc.

[43] Alzoubi H, Alzubi R, Ramzan N, West D, Al-Hadhrami T, Alazab M. A Review of Automatic Phenotyping Approaches using Electronic Health Records. Electronics. 2019;8(11).

