# Supplementary material for: Development and validation of algorithms to identify patients with chronic kidney disease and related chronic diseases across the Northern Territory, Australia
Source: BMC Nephrol. 2022 Sep 23;23:320. doi: 10.1186/s12882-022-02947-9 (PMC9502610; doi:10.1186/s12882-022-02947-9)

Supplemental Material

Manuscript: Development and validation of algorithms to identify patients with chronic kidney disease and related chronic diseases across the Northern Territory, Australia

Authors: Winnie Chen, Asanga Abeyaratne, Gillian Gorham, Pratish George, Vijay Karepalli, Dan Tran, Christopher Brock, Alan Cass, on behalf of the Territory Kidney Care Steering Committee

Supplemental Table 1: Key algorithm assumptions for CKD sub-phenotypes

Supplemental Table 2: Inter-reviewer agreement and reliability measures

Supplemental Table 3: Data metrics for all active TKC patients

Supplemental Table 4: Territory Kidney Care database completeness metrics

Supplemental Table 5.1: 2x2 Table for CKD phenotype (CKD stage 1 or higher)

Supplemental Table 5.2: 2x2 Table for T2DM phenotype

Supplemental Table 5.3: 2x2 Table for hypertension phenotype

Supplemental Table 5.4: 2x2 Table for cardiovascular disease phenotype

Supplemental Figure 1: ROC curve for CKD phenotype (stage 1 or higher)

Supplemental Figure 2: ROC curve for T2DM phenotype

Supplemental Figure 3: ROC curve for hypertension phenotype

Supplemental Figure 4: ROC curve for cardiovascular disease phenotype

Supplemental Table 1: Key algorithm assumptions for CKD sub-phenotypes

		**Albuminuria categories (mg/mmol)**		
		**A1**<br>**<3**	**A2**<br>**3-30mg/mmol**	**A3**<br>**>30mg/mmol**
**eGFR categories ($mL/min/1.73m^2$)**	**G1**	No CKD	**Sub-phenotype: CKD stage 1**<br>eGFR >90 AND 1 or more uACR >3 within the past 2 years	
	**G2**	No CKD	**Sub-phenotype: CKD stage 2**<br>2 or more eGFR 60-89 >3 months apart AND 1 or more uACR >3 within the past 2 years	
	**G3a**	**Sub-phenotype: CKD stage 3a**<br>eGFR 45-59 for >3 months		
	**G3b**	**Sub-phenotype: CKD stage 3b**<br>eGFR 30-44 for >3 months		
	**G4**	**Sub-phenotype: CKD stage 4**<br>eGFR 15-29 for >3 months		
	**G5**	**Sub-phenotype: CKD stage 5**<br>eGFR <15 for >3 months		
	**RRT (haemodialysis*, peritoneal dialysis, transplant)**	**Sub-phenotype: RRT**<br>Presence of one or more: administrative codes (ICD/ICPC) or procedural codes associated with RRT of any type.		

Abbreviations: eGFR – estimated glomerular filtration rate; ICD – International Classification of Disease; ICPC – International Classification of Primary Care; RRT – renal replacement therapy; uACR – urine albumin-to-creatinine ratio

*>10 haemodialysis procedural codes in a 12-month timeframe is required to meet the haemodialysis phenotype

Supplemental Table 2: Inter-reviewer agreement and reliability measures

	**% Agreement**	**Cohen's Kappa**	**95% CI**
CKD	83.51	0.8026	0.7560-0.8877
T2DM	94.44	0.8616	0.7954-0.9277
Hypertension	82.99	0.6622	0.5768-0.7476
Cardiovascular disease	91.67	0.7837	0.7008-0.8665

Abbreviations: CI – Confidence interval T2DM – Type 2 diabetes mellitus

Supplemental Table 3: Data metrics for all active TKC patients

	**Entry recorded (%) (total n=48,569 active patients*)**	**Median number of results per patient (n, IQR)**	**Median number of results per patient per year (n, IQR)**
**ICD code**	30,956 (64%)	3 (0-12)	0.2 (0-1.0)
**ICPC code**	30,599 (64%)	3 (0-8)	0.2 (0-0.5)
**Medication entries**	45,852 (94%)	18 (0-66)	1.3 (0-4.0)
**Observation entries**	32,180 (66%)	26 (0-91)	1.9 (0-5.4)
**Laboratory results**	33,172 (68%)	116 (40-261)	9.0 (4.0-18.6)

Abbreviations: IQR – Interquartile range; ICD – International Classification of Disease; ICPC – International Classification of Primary Care

*Active is defined as patients with a TKC database entry within the past 2 years.

Supplemental Table 4: Territory Kidney Care database completeness metrics

**Metric**	**Completeness metric**	**Of all active within last 2 years (n=48569)**
Metric 1	3 or more laboratory results OR<br>3 or more observation entries	46025 (94.76%)
Metric 2	3 or more laboratory results AND<br>3 or more observation entries	31609 (65.08%)
Metric 3	3 or more laboratory results AND<br>3 or more observation entries AND<br>1 or more ICD/ICPC code	30411 (62.61%)
Metric 4	3 or more laboratory results AND<br>3 or more observation entries AND<br>1 or more ICD/ICPC code AND<br>1 or more medication entry	29593 (60.93%)

Abbreviations: ICD – International Classification of Disease; ICPC – International Classification of Primary Care

Supplemental Table 5.1: 2x2 Table for CKD phenotype (CKD stage 1 or higher)

Tables 5.1 to 5.4 report diagnostic accuracy of Territory Kidney Care (TKC) algorithms versus clinician chart review (gold standard). Sensitivity, specificity, positive predictive value (PPV), negative predictive value (NPV), and ROC area under curve (ROC AUC) are reported with 95% confidence intervals (95%CI).

	**Disease + (Clinician)**	**Disease – (Clinician)**	**Total**
**Disease + (TKC algorithm)**	182	25	207
**Disease – (TKC algorithm)**	14	67	81
**Total**	196	92	288

Sensitivity = 92.86% (95%CI 89.25 to 96.46%)

Specificity = 72.83% (95%CI 63.74 to 81.92%)

NPV = 82.72% (95%CI 74.48 to 90.95%)

PPV = 87.92% (95%CI 83.48 to 92.36%)

ROC AUC = 0.8284 (95%CI 0.7793 to 0.8776)

Supplemental Table 5.2: 2x2 Table for T2DM phenotype

	**Disease + (Clinician)**	**Disease – (Clinician)**	**Total**
**Disease + (TKC algorithm)**	60	7	67
**Disease – (TKC algorithm)**	20	201	221
**Total**	80	208	288

Type 2 diabetes mellitus phenotype – definition did not include type 1 diabetes mellitus, or hyperglycaemia in pregnancy

Sensitivity = 75.00% (95%CI 65.51 to 84.49%)

Specificity = 96.63% (95%CI 94.18 to 99.09%)

PPV = 89.55% (95%CI 82.23 to 96.88%)

NPV = 90.95% (95%CI 87.17 to 94.73%)

ROC AUC = 0.8592 (95%CI 0.8089 to 0.9075)

Supplemental Table 5.3: 2x2 Table for hypertension phenotype

	**Disease +<br><br>(Clinician)**	**Disease –<br><br>(Clinician)**	**Total**
**Disease +<br><br>(TKC algorithm)**	122	17	139
**Disease –<br><br>(TKC algorithm)**	21	128	149
**Total**	143	145	288

Sensitivity = 85.31% (95%CI 79.51 to 91.12%)

Specificity = 88.28% (95%CI 83.04 to 93.51%)

PPV = 87.77% (95%CI 82.32 to 93.22%)

NPV = 85.91% (95%CI 80.32 to 91.49%)

ROC AUC = 0.8680 (95%CI 0.8287 to 0.9072)

Supplemental Table 5.4: 2x2 Table for cardiovascular disease phenotype

	**Disease +<br>(Clinician)**	**Disease –<br>(Clinician)**	**Total**
**Disease +<br>(TKC algorithm)**	61	8	69
**Disease –<br>(TKC algorithm)**	16	203	219
**Total**	77	211	288

Cardiovascular disease phenotype – pooled phenotype, definition included ischaemic heart disease, atrial fibrillation, valvular heart disease, peripheral vascular disease, and cerebrovascular disease

Sensitivity = 79.22% (95%CI 70.16 to 88.28%)

Specificity = 96.21% (95%CI 93.63 to 98.79%)

PPV = 88.41% (95%CI 80.85 to 95.96%)

NPV = 92.69% (95%CI 89.25 to 96.14%)

ROC AUC = 0.8771 (95%CI 0.8297 to 0.9246)

Supplemental Figure 1: ROC curve for CKD phenotype (CKD stage 1 or higher)

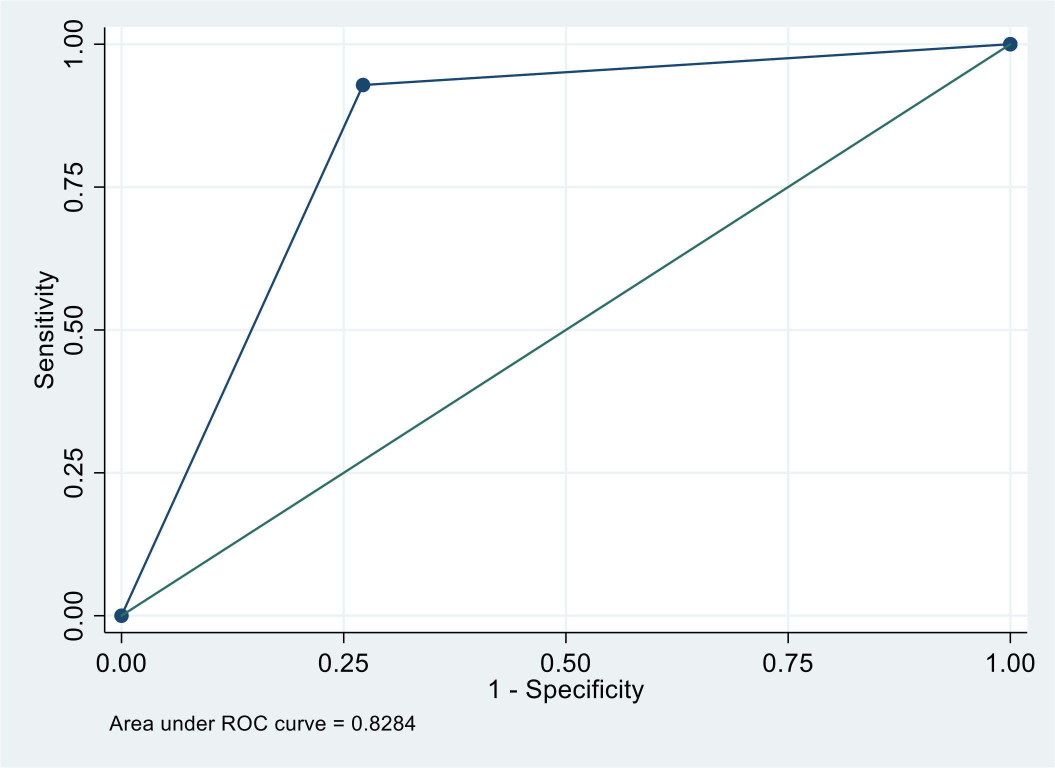

Supplemental Figure 2: ROC curve for T2DM phenotype

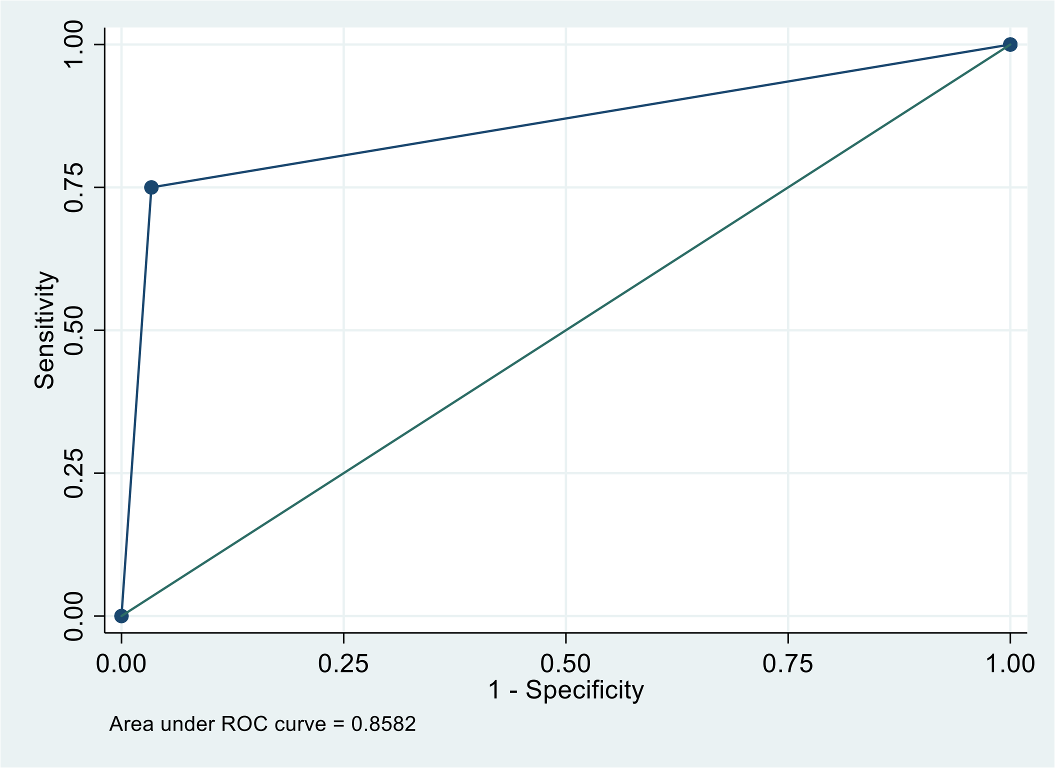

Supplemental Figure 3: ROC curve for hypertension phenotype

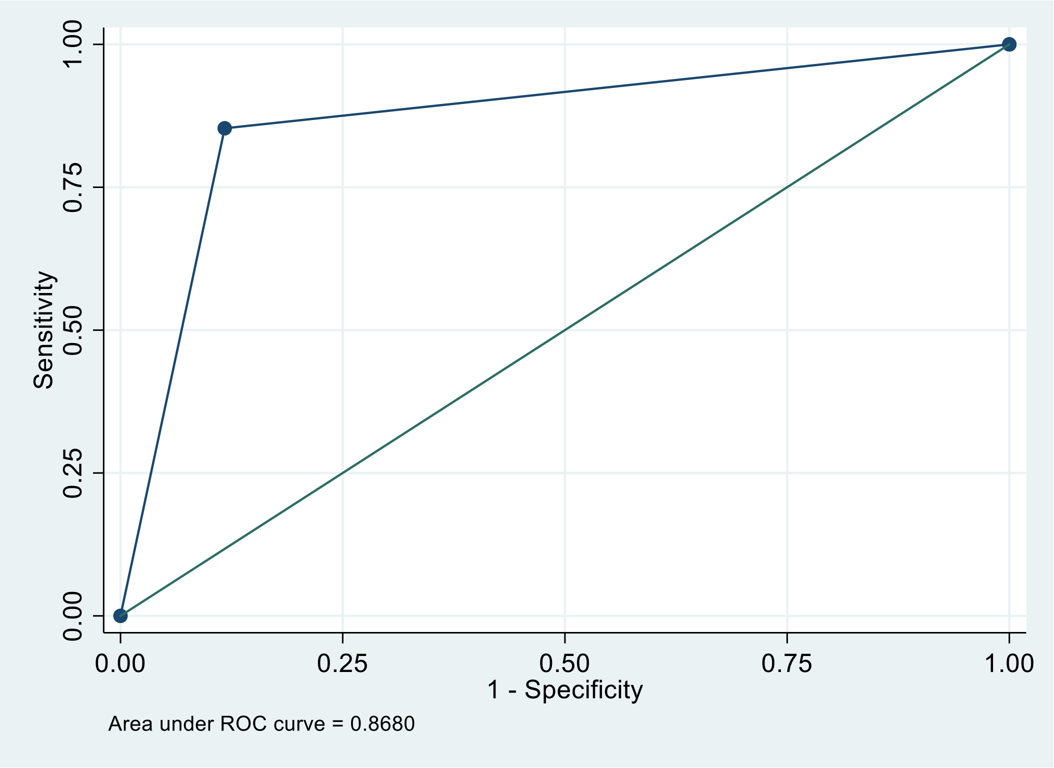

Supplemental Figure 4: ROC curve for cardiovascular disease phenotype

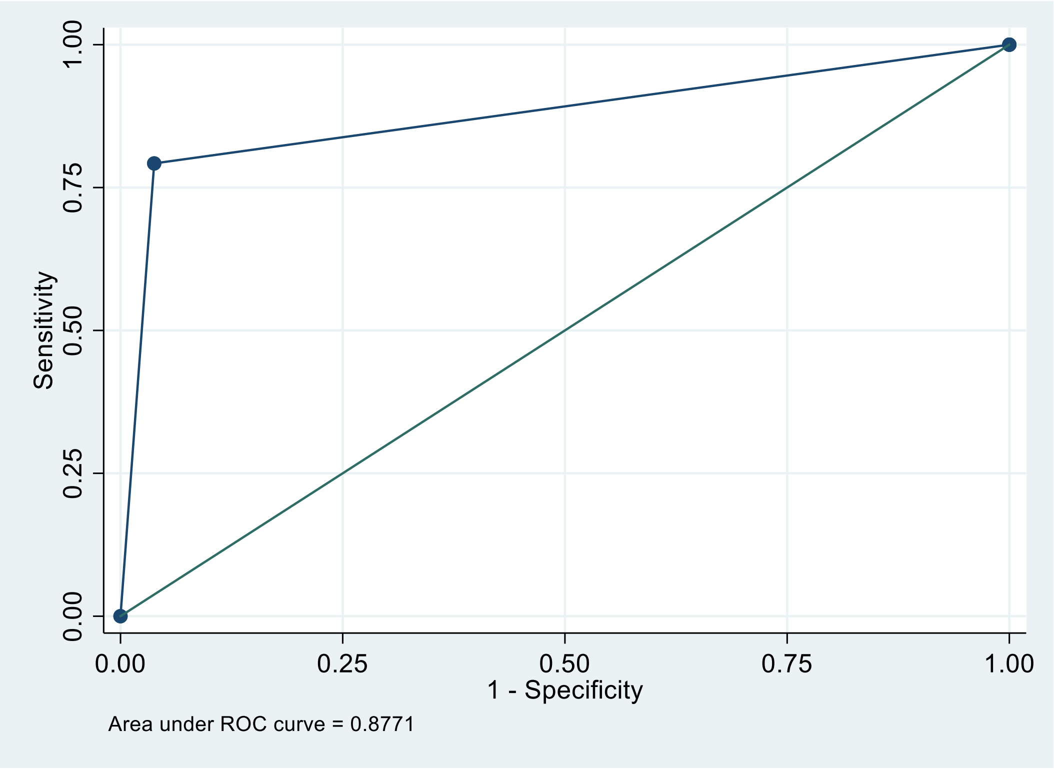

Supplement: Supplementary file 1 — Additional file 1. [file 12882_2022_2947_MOESM1_ESM.pdf]
